# Adaptive optics two-photon microscopy enables near-diffraction-limited and functional retinal imaging in vivo

**DOI:** 10.1038/s41377-020-0317-9

**Published:** 2020-05-06

**Authors:** Zhongya Qin, Sicong He, Chao Yang, Jasmine Sum-Yee Yung, Congping Chen, Christopher Kai-Shun Leung, Kai Liu, Jianan Y. Qu

**Affiliations:** 1 Department of Electronic and Computer Engineering, The Hong Kong University of Science and Technology, Clear Water Bay, Kowloon, Hong Kong, China; 2 Division of Life Science, The Hong Kong University of Science and Technology, Clear Water Bay, Kowloon, Hong Kong, China; 3 Department of Ophthalmology and Visual Sciences, The Chinese University of Hong Kong, Hong Kong, China; 4 State Key Laboratory of Molecular Neuroscience, The Hong Kong University of Science and Technology, Clear Water Bay, Kowloon, Hong Kong, China; 5 Center of Systems Biology and Human Health, The Hong Kong University of Science and Technology, Clear Water Bay, Kowloon, Hong Kong, China

**Keywords:** Biophotonics, Adaptive optics, Multiphoton microscopy

## Abstract

In vivo fundus imaging offers non-invasive access to neuron structures and biochemical processes in the retina. However, optical aberrations of the eye degrade the imaging resolution and prevent visualization of subcellular retinal structures. We developed an adaptive optics two-photon excitation fluorescence microscopy (AO-TPEFM) system to correct ocular aberrations based on a nonlinear fluorescent guide star and achieved subcellular resolution for in vivo fluorescence imaging of the mouse retina. With accurate wavefront sensing and rapid aberration correction, AO-TPEFM permits structural and functional imaging of the mouse retina with submicron resolution. Specifically, simultaneous functional calcium imaging of neuronal somas and dendrites was demonstrated. Moreover, the time-lapse morphological alteration and dynamics of microglia were characterized in a mouse model of retinal disorder. In addition, precise laser axotomy was achieved, and degeneration of retinal nerve fibres was studied. This high-resolution AO-TPEFM is a promising tool for non-invasive retinal imaging and can facilitate the understanding of a variety of eye diseases as well as neurodegenerative disorders in the central nervous system.

## Adaptive-Optics Fluorescence Microscopy: Seeing clearly into eyes

An advance in Two-Photon Excitation Fluorescence Microscopy (TPEFM) allows greatly improved subcellular imaging of the retina, promising better understanding of the eye and the central nervous system in health and disease. Researchers led by Jianan Y. Qu at Hong Kong University of Science and Technology used adaptive optics techniques to correct the optical aberrations that have limited application of TPEFM in living eyes. They demonstrated their innovation using fluorescent detector molecules to image the activity of calcium ions in mouse retinal neurons. They also monitored subcellular structures in microglial cells, and guided laser light to cut the axons that transmit nerve signals. This technology could help investigate the development of neurodegenerative diseases, since the eye offers a window into nerves of the central nervous system that link the eye with the brain.

## Introduction

As a window to the brain, the retina is the only part of the central nervous system (CNS) that can be visualized non-invasively via optical imaging. Accumulating evidence shows that the eye can host immune responses similar to those in the brain and spinal cord and that ocular symptoms always precede the traditional diagnosis of CNS pathologies, such as Alzheimer’s disease, Parkinson’s disease and multiple sclerosis^1,2^. Therefore, in vivo morphological and functional imaging of retinal structures provides a non-invasive approach to not only understand eye diseases but also decipher the mechanisms behind neurodegenerative disorders in the CNS. Over the past decades, fluorescence imaging of the retina in small animal models has become increasingly important for delineating the pathophysiological characteristics implicated in a spectrum of diseases^3–5^. Among the emerging optical imaging tools, two-photon excitation fluorescence microscopy (TPEFM) offers unique benefits for retinal imaging. Most importantly, near-infrared excitation lasers do not cause any crosstalk with visible stimulation, making TPEFM desirable for the functional imaging of retinal neurons and neuronal networks. In addition, TPEFM based on near-infrared excitation is particularly well suited to simultaneously excite multiple fluorophores in the retina because the eye is optically transparent to long-wavelength light. Furthermore, as the dilated pupil of rodents has a much larger numerical aperture (NA) (~0.5) than that of humans or primates (<0.2)^6,7^, two-photon imaging of the mouse eye could achieve a high spatial resolution.

However, optical aberrations of the mouse eye distort the wavefront of the excitation laser and deteriorate the imaging quality^6,8^. Compensation of ocular aberrations and recovery of a diffraction-limited point spread function are of particular importance for TPEFM because the efficiency of two-photon excitation is proportional to the square of the incident light intensity. Adaptive optics (AO), an optical technique originally implemented in astronomical telescopes, has recently been employed to correct ocular aberrations and improve the resolution for in vivo two-photon retinal imaging in animal models^9–15^. Compared to the sensorless AO technique^14–17^, which adopts image-based iteration algorithms to optimize the signal intensity or image contrast, the direct wavefront-sensing method can usually achieve faster AO correction to image highly dynamic biological structures or processes in the retina^18^.

In this work, we advance the AO-TPEFM method using direct wavefront measurement of a nonlinear fluorescent guide star^19–21^ for near-diffraction-limited and functional retinal imaging in living mice. In particular, an electrically tuneable lens (ETL) was employed to countervail the exceptionally large optical power (>500D^6^) of the mouse eye and to finely tune the focal plane of two-photon imaging through different retinal layers of interest. We built a wavefront sensor equipped with an ultrasensitive electron-multiplying charge-coupled device (EMCCD) camera for wavefront sensing, enabling accurate and fast measurement of ocular aberrations based on the weak two-photon fluorescence guide-star signals in the imaging plane. Benefiting from the recovery of near-diffraction-limited resolution after AO correction, the fine structures in different retinal layers could be clearly resolved, and simultaneous calcium imaging of the somas and dendrites of retinal ganglion cell (RGCs) was demonstrated. In addition, it is shown that AO-TPEFM is capable of visualizing the fine processes of retinal microglia and resolving their structural alterations in the pathological retina. Finally, for the first time, precise laser microsurgery of retinal nerve fibres was achieved with the aid of AO, enabling longitudinal study of RGC axonal degeneration in vivo. By imaging the morphological and functional dynamics of various retinal structures with high spatial and temporal resolution, this technique offers great opportunities to disclose the mechanisms of eye diseases and evaluate therapeutic strategies with high fidelity.

## Results

### In vivo retinal imaging at subcellular resolution

**Fig. 1:**
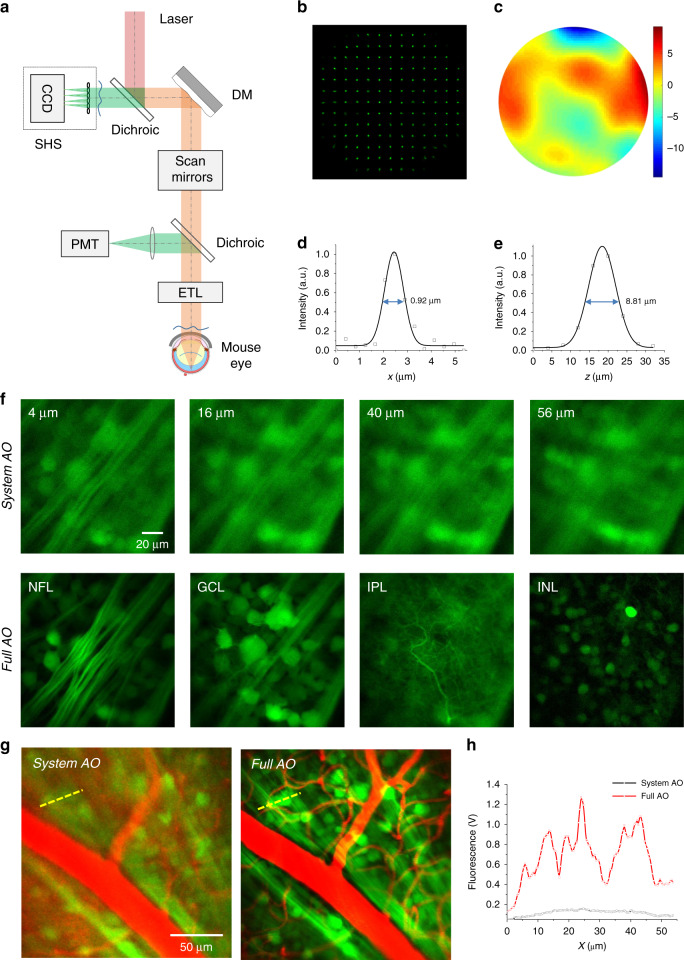
AO-TPEFM enables non-invasive subcellular imaging of the mouse retina. **a** Schematics of in vivo AO-TPEFM of the mouse retina using direct wavefront sensing from a two-photon fluorescence guide star. **b** Representative spot diagram collected by the SHWS. **c** Representative aberration wavefront of the mouse eye (unit: μm). **d**, **e** The lateral (**d**) and axial (**e**) resolution of the AO-TPEFM system after full AO correction. The mean values of the measured FWHM from six locations are shown in the figures. **f** Depth-resolved imaging of different retinal layers with system (top) or full (bottom) AO correction. **g** Mosaic TPEF images of RGCs (green) and blood vessels (red) with system (left) and full (right) AO correction. **h** Signal profiles along the dashed lines in (**g**) for a comparison of the fluorescence intensity of RGCs with system (red line) and full (black line) AO correction

An AO-TPEFM system based on direct wavefront sensing was developed for in vivo fluorescence imaging of the mouse retina (Figs. 1a and S1). In particular, the lens of the mouse eye functions as an inherent objective to focus the excitation light into the retina and collect emission fluorescence signals. Moreover, since the mouse retina is over 50 times thicker than the human eye in dioptres due to the especially high optical power of the mouse eye^6^, an ETL with a large tuning range was used to quickly section different retinal layers. The ETL and the group of relay lenses formed a compact assembly with optimized optical performance (Figs. S2 and S3), allowing for seamless integration into standard fluorescence microscopes. Guide star signals, the descanned two-photon fluorescence from the retina, were collected by a custom-built Shack-Hartmann wavefront sensor (SHWS) consisting of a high-sensitivity EMCCD camera and a microlens array. The ultrasensitive wavefront sensor and the optimized optical design maximize the fluorescence collection efficiency for both wavefront sensing and two-photon imaging, thus enabling fast aberration measurement and AO correction. The spot pattern obtained by the SHWS (Fig. 1b) was then analysed with a weighted least squares algorithm to reconstruct the corrective wavefront (Fig. 1c), and the decomposed Zernike polynomials were then used to control a high-speed deformable mirror (DM) in a closed-loop configuration (Fig. S4). The ocular aberrations originate not only from the optics of the mouse eye but also from the contact lens (Fig. S5), which was used as a protective element to maintain cornea hydration and prevent cataracts during retinal imaging^22^. To quantify the resolution of AO-TPEFM for in vivo retinal imaging, we measured the cross-sections of the fine processes of microglia (Figs. 1d, e and S6). The lateral and axial resolutions of AO-TPEFM estimated from the full width at half maximum (FWHM) of the line and point spread functions^6^ are 0.92 ± 0.05 μm and 8.81 ± 1.07 μm, respectively, close to the diffraction limit (0.71 and 6.16 μm calculated for the 920 nm laser and 0.49 excitation NA, respectively^7,23,24^). Given that it is unknown whether the sizes of the targeted microglial processes are smaller than the diffraction limit, the estimation of the resolution of the AO-TPEFM system is conservative.

We first performed in vivo morphological imaging of the mouse eye that was intravitreally injected with AAV2-hsyn-GFP to label retinal neurons with green fluorescent protein (GFP). The results show that with system AO correction alone, the fluorescence images are severely blurred (Fig. 1f). After full correction of both the system and eye-induced aberrations, the depth-resolved fluorescence images reveal the fine structures of neurons and processes in different retinal layers, thanks to the substantially improved spatial resolution (Figs. 1d, e and S6). Specifically, the axons and somas of RGCs are visualized in the nerve fibre layer (NFL) and the ganglion cell layer (GCL). Moreover, the individual dendrites of RGCs are finely resolved in the inner plexiform layer (IPL). At deeper locations, neurons such as bipolar cells and amacrine cells are clearly visualized in the inner nuclear layer (INL)^25^. In addition, coupled with angiography using fluorescent contrasts^26^, fine structures of microvascular and neuronal networks in the retina can be visualized simultaneously (Figs. 1g and S7). Thanks to the drastically enhanced fluorescence intensity and improved image contrast (Figs. 1h and S7), the retinal blood flow velocity can be precisely measured in vivo (Fig. S8), which enables functional assessment of retinal metabolism^27^.

### Simultaneous functional calcium imaging of RGC somas and dendrites

**Fig. 2:**
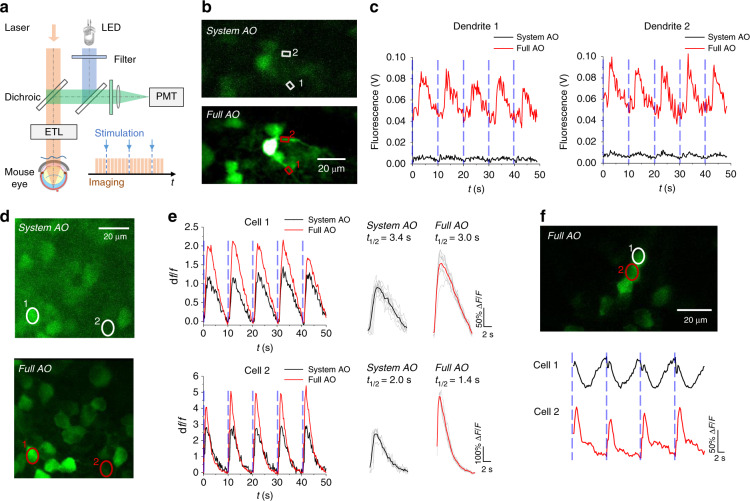
In vivo functional calcium imaging of RGCs. **a** Schematics for the calcium imaging experiment. **b** Two-photon imaging of RGCs labelled with AAV9-hsyn-GCaMP6s. Dendrites are finely resolved after full AO correction. **c** Calcium transients of RGC dendrites marked in (**b**). The vertical dashed lines indicate the timing of blue light flash stimulation. **d** Two-photon fluorescence images of the GCaMP-labelled RGCs with the system and full AO correction. **e** Calcium response of cells of interest (marked in (**d**)) with system and full correction. The left plane indicates the change in fluorescence intensity relative to the resting fluorescence intensity (∆f/f), and the right plane shows the averaged calcium response and the decay half-life. **f** Calcium imaging with full AO correction reveals different types of RGCs

Because functional alterations of RGCs precede structural changes in retinal neurodegenerative diseases^28,29^, calcium imaging of RGCs in response to visible light provides opportunities for early detection of neuron pathology^30,31^. In particular, recent studies have suggested that the dendrites of RGCs, which receive synaptic inputs from presynaptic bipolar and amacrine cells, have diverse calcium signalling pathways that likely contribute to differences in the visual function of RGCs^32^. Although a recent study demonstrated two-photon calcium imaging of the mouse retina without AO, only the visual responses of somas were measured, while the dendritic calcium signals were not visualized clearly due to limited imaging resolution^33^. Here, we measured the light-evoked calcium responses of RGCs labelled by intravitreal injection of AAV9-hsyn-GCaMP6s (Fig. 2a). Benefiting from the high spatial resolution of AO-TPEFM, dendritic arbors of RGCs are clearly resolved, allowing for simultaneous recording of the somatic and dendritic calcium signals (Figs. 2b, c, S9 and Video S1). The results demonstrate that the primary dendrites show stronger and faster responses to blue light flash than the neuronal soma (Fig. S9). Specifically, the decay half-lives (*t*_1/2_) calculated from a single exponential fitting are 3.0, 2.4 and 2.2 s for the soma and two dendrite segments (Fig. S9). As demonstrated, full AO correction is indispensable for accurate interpretation of calcium responses, including the change in fluorescence intensity and the decay half-lives (Fig. 2d, e, Video S2). Furthermore, a diversity of calcium dynamics was observed among distinct functional types of RGCs, such as ON and OFF RGCs (Fig. 2f, Video S3), of which the calcium signalling pathways encode the onset and offset of light, respectively^34,35^. Given that the light response properties among RGCs are differentially impaired in the glaucomatous retina^36^, in future work, it would be of great interest and value to study the heterogeneity of functional alterations across the somas and dendrites of RGCs in order to comprehensively evaluate the pathophysiology of glaucoma in terms of RGC excitability and neurotransmission.

### Time-lapse characterization of microglial structure and dynamics

**Fig. 3:**
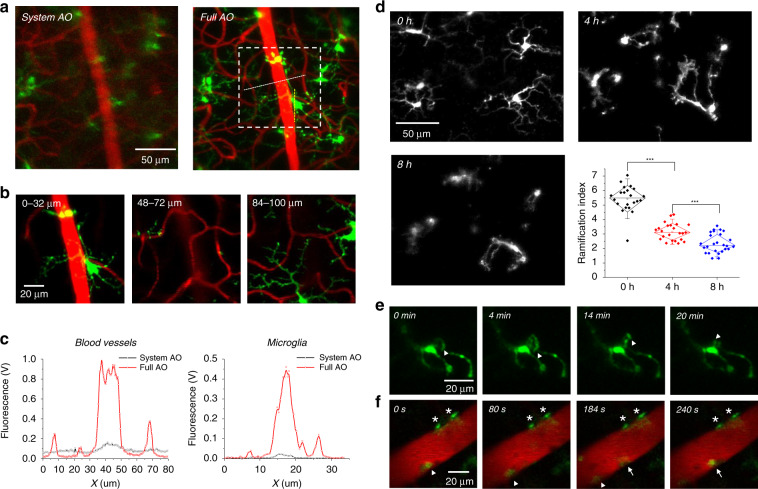
Study microglial dynamics and function in the mouse retina. **a** Mosaic TPEF projection images of microglia (green) and blood vessels (red) with system and full AO correction in the retina of Cx3Cr1-GFP transgenic mice. **b** Enlarged images of microglia and vascular structures in different retinal layers corresponding to the white dashed box in (**a**). **c** Signal intensity profiles of blood vessels and microglia along the dashed lines in (**a**). **d** Time-lapse images of retinal microglia and blood vessels before NMDA administration and four and eight hours after NMDA administration. The lower right corner shows the change in the ramification index of retinal microglia in response to NMDA administration. Each time point involves more than 20 microglia from three mice. **e** Time-lapse imaging showing the dynamics of the microglial process four hours after NMDA administration. The arrowheads mark the mobility of a microglial process. **f** Representative two-photon images showing the recruitment of round leukocytes eight hours after NMDA treatment. Arrowheads and arrows: two GFP^+^ leukocytes migrating along a retinal blood vessel; asterisks: two microglia attached on the endothelia of the blood vessel

Microglia, the resident immune cells in the CNS, play central roles in the regulation of neuroinflammation. Due to their high sensitivity to subtle alterations in the microenvironment, in vivo imaging and analysis of microglial structures is crucial to the study of the early onset and development of retinal diseases^37^. Recent studies have revealed transient microglial migration and recruitment following retinal injury and neuroinflammatory diseases through longitudinal imaging of the mouse retina^38,39^. The results suggested intensive involvement and dynamic behaviour of microglia during retinal remodelling. In our study, the improved resolution of the AO-TPEFM system allowed us to characterize the dynamics of the fine processes of microglia and thus could provide direct evidence to identify early microglial activation in response to pathogens. Therefore, we applied AO-TPEFM imaging to study the morphological characteristics of microglia at high spatiotemporal resolution in Cx3Cr1-GFP transgenic mice. We demonstrated that after full AO correction, the branching processes of microglia can be clearly visualized at various retinal depths in the GCL, IPL and outer plexiform layers^40^ (Fig. 3a–c). N-methyl-D-aspartic acid (NMDA) excitotoxicity is considered to contribute to RGC loss in glaucoma^41^. Nonetheless, how NMDA affects microglial phenotypes remains largely unexplored, particularly in vivo. Taking advantage of the high-resolution AO-TPEFM, we conducted a longitudinal study of microglial activation in the mouse eye with intraocular injection of NMDA, which is an acute model of retinal damage^42,43^. Time-lapse imaging revealed morphological alterations in microglia four and eight hours after NMDA administration (Fig. 3d). Specifically, the microglia in the NMDA-administered eyes exhibited a decreased ramification index (Figs. 3d and S10), which is a typical characteristic of reactive microglia^44,45^. By virtue of the fast AO correction, the morphological dynamics of retinal microglia can be monitored with high resolution (Fig. 3e, Video S4). In addition, with the aid of AO, round leukocytes were observed to migrate to the retina through blood vessels after NMDA treatment (Fig. 3f, Video S5), also indicating the activation of inflammatory responses. Given that microglial activation often precedes reactions of other cell types in the CNS^46^, the non-invasive detection of microglial morphologies by AO-TPEFM may facilitate the diagnosis of CNS disorders at an early stage.

### Laser axotomy on retinal nerve fibres

**Fig. 4:**
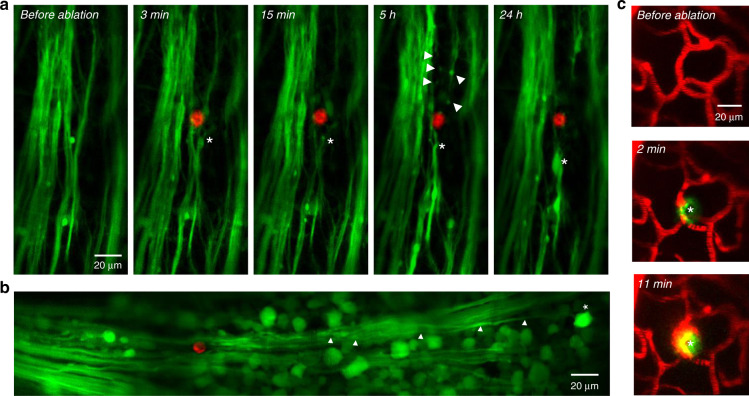
AO enables imaging-guided laser microsurgery in the mouse retina. **a** Laser axotomy for tracing axonal degeneration. Green: nerve fibres of RGCs labelled by AAV2-hsyn-GFP; red: fluorescence signal produced by laser ablation; asterisk: swelling and retraction of proximal axons; arrowheads: segmentation of distal axons. **b** High-precision laser axotomy for tracing the axonal degeneration of a single RGC. Arrowheads show the degenerated axon of the RGC marked by the asterisk. **c** Laser ablation of retinal microvasculature. Red, blood vessel; green, fluorescence signal produced by laser ablation; asterisk, laser ablation site

The optic nerve, composed of the axons of RGCs, transmits visual information from the retina to the lateral geniculate nucleus in the thalamus and the superior colliculus in the midbrain. Axonal degeneration in RGCs has been regarded as one of the most important early signs of glaucomatous optic neuropathy and may result in devastating consequences such as progressive RGC death and irreversible vision loss^47–50^. Recently, the femtosecond laser was used as a powerful tool for imaging-guided microsurgery in a host of biological tissues and can produce unique fluorescent compounds that can serve as a contrast agent to visualize the microsurgery boundary^51,52^. Therefore, we conducted laser microsurgery on nerve fibres and characterized axonal degeneration by repeatedly imaging the injured axons in the NFL. With system AO correction only, the high-power femtosecond laser could not induce axonal injury in the retina, likely because of the distorted laser focus. After correcting all the aberrations, however, spatially confined laser ablation could be conducted in the NFL of the retina, and time-lapse imaging revealed the degeneration process of the injured nerve fibres (Fig. 4a). In contrast to the traditional traumatic optic neuropathy models using optic nerve crush or transection^53,54^, this imaging-guided laser microsurgery technique can either enable high-precision laser axotomy for tracing the axonal degeneration of a single RGC or produce a large lesion on a number of nerve fibre bundles of interest (Figs. 4b and S11a). In addition, laser microsurgery can also be performed on the retinal microvasculature (Figs. 4c and S11b), providing an in vivo model to study retinal vessel injury and repair. In combination with advanced axonal protection approaches, this microsurgery technique may enhance our understanding of the mechanisms underlying axonal growth at the single-cell level and may shed light on novel treatment strategies for neurodegenerative diseases^54^.

## Discussion

We have shown that the high spatial resolution and 3D sectioning capability of AO-TPEFM permits subcellular retinal imaging through the pupil of the mouse eye. Taking advantage of advances in fluorescent labelling and mouse models of human disease, AO-TPEFM can potentially be used to unveil the correlation among vascular defects, RGC death, axon damage and microglial activation in the pathological retina^55^, which will facilitate a more accurate prognosis and open new doors to potential treatments.

It has been reported that monochromatic aberrations substantially change with depth in the mouse retina^56^, consistent with our findings that the AO correction effect would be compromised if the wavefront is measured far away from the imaging plane (Fig. S12). Therefore, direct wavefront sensing based on nonlinear fluorescent guide stars would be advantageous for accurate measurement of ocular aberrations at the exact imaging location and thus permits highly efficient AO correction. Currently, the imaging speed of AO-TPEFM is mainly limited by the wavefront measurement, which takes 0.1–2 s depending on the fluorescence intensity of guide stars. We found that the aberrations measured from a fixed position in the retina are stable over a sufficiently long period of time (Fig. S13), suggesting that even weak fluorescence could be used as guide stars for AO correction by increasing the exposure time of the wavefront sensor.

It must be emphasized that many groups have explored in vivo AO retinal imaging using back-scattered light as guide stars and obtained retina images of high quality^6,57,58^. The technology shows superior performance in at least two aspects. First, the strong signals of guide stars enable near video-rate AO correction^59,60^, which is minimally affected by eye movements occurring randomly during imaging. Second, wavefront sensing does not require any fluorescent label, which is of great benefit for human retinal imaging and clinical applications^61–65^. However, the aberration measured with the back-scattered light from the outer retina may not be perfectly applicable to the inner retina^56^. For single-photon scanning laser ophthalmoscopy, the excitation source causes cross-talk in the functional imaging of retinal neurons responding to the stimulation of visible light signals^30^.

In this study, we did not observe retinal injury under the current laser power used for wavefront sensing and two-photon imaging. Future work could be focused on improving the AO algorithm and detection efficiency to reduce the laser power required for AO correction and imaging. Previous works demonstrated that the laser power for two-photon imaging of the retina can be reduced by applying image registration and averaging methods^66^ or modulating the ultrashort laser pulses with dispersion compensation^67,68^. Pioneering studies on safety assessments for two-photon imaging of the retina provide guidance to ensure that our AO-TPEFM can safely obtain high-resolution retinal images^69–71^. Furthermore, combined with fluorescence lifetime imaging of the retina, AO-TPEFM may provide great opportunities to study the biochemistry of retinal neurons and the visual cycle and offer insight into cell-microenvironment interactions in the retina^72–74^.

## Materials and methods

### Adaptive optics two-photon microscopy

A schematic of the AO two-photon excitation fluorescence microscopy (AO-TPEFM) system is shown in Figs. 1a and S1. Briefly, two femtosecond lasers (Coherent, Mira 900) tuned at 920 nm and 740 nm were expanded to slightly overfill the aperture of the deformable mirror (DM, Alpao, DM97-15) and combined using a polarizing beamsplitter. The laser beam reflected by the DM was de-magnified threefold by a pair of achromatic relay lenses L5 and L6 (focal lengths: 225 mm and 76.2 mm) to match the 5-mm galvanometer scan mirrors. The galvo X and Y mirrors (Cambridge Technology, 6215H) were conjugated by a 4-f telescope formed by another pair of achromatic relay lenses L7 and L8 (focal lengths: 100 mm and 100 mm). Then, the galvo Y mirror was relayed to the electrically tuneable lens (ETL, Optotune, EL-16-40-TC-VIS-5D-C) by lens pair L9 and L10 (focal lengths: 75 mm and 250 mm). A 4-f system composed of lens pair L15 and L16 (focal lengths: 100 mm and 13.5 mm) relayed the ETL to the mouse cornea and compressed the excitation beam to approximately 2 mm in diameter. The ETL and relay lens system were designed to be an add-on module (Fig. S1) that can be easily incorporated into standard microscope systems for in vivo retinal imaging through the mouse pupil. The aberrations of this module were analysed with Zemax by replacing the mouse eye with a perfect lens. As shown in Fig. S3, this module has diffraction-limited performance in the vergence range from −50 to 50 dioptres over a 200-µm field of view (FOV) for retinal imaging.

For two-photon fluorescence imaging, the fluorescence emission signals were reflected by a dichroic mirror D2 (Semrock, FF705-Di01-25×36) and further separated by another dichroic mirror D3 (Semrock, FF560-Di01-25×36) into green and red channels. Appropriate bandpass and short-pass filters were placed in front of the photomultiplier tubes (Hamamatsu, H11461-01 and H11461-03) to reject the excitation laser and select particular wavelength ranges of detection.

For wavefront sensing, D2 was replaced with another dichroic mirror (Semrock, Di02-R488-25×36). The emitted fluorescence signal followed the reverse path of the excitation and was descanned by the galvo mirrors and reflected by the DM before being separated from the femtosecond laser by dichroic mirror D1 (Semrock, FF705-Di01-25×36). Then, the two-photon fluorescence guide star was de-magnified twice by lens pair L11 and L12 (focal lengths: 200 mm and 100 mm) to match the aperture of the custom-built Shack-Hartman wavefront sensor (SHWS) consisting of a microlens array (SUSS MicroOptics, 18-00197) and an electron-multiplying charge-coupled device (Andor iXon3 888). The microlens array, DM, two galvo mirrors, ETL and mouse cornea were all mutually conjugated.

### System calibration

Following previously reported procedures^21^, the DM was first calibrated separately using a Michaelson interferometer. The influence matrix of DM actuators was obtained by applying a voltage to each actuator one by one and measuring the wavefront change in terms of Zernike modes. Then, the voltage patterns driving the first 65 Zernike modes could be calculated by taking the generalized inverse of the influence matrix. In this way, we can generate any desired wavefront using a linear combination of those 65 modes.

For system AO calibration, the compact ETL and relay lens module were replaced by an objective (Olympus, XLPLN25XWMP2, 1.05 NA). The SHWS was calibrated with the DM in the system to minimize alignment error. First, the fluorescent dye solution (rhodamine 6G) was placed at the focal plane of the microscope, and the two-photon excitation process created a fluorescent guide star at the focus of the objective lens. Light from the guide star was descanned by the galvo mirrors and reflected by the DM before directing to the SHWS. Therefore, the wavefront change of the DM could be detected by the SHWS. Then, the first 65 Zernike modes were applied to the DM sequentially, and the corresponding displacement of spots on the SHWS was measured. These SHWS measurements M_sz_ represent the linear mapping of DM Zernike modes $\Delta {\mathrm{Z}}_{{\mathrm{DM}}}$and SHWS spot shift $\Delta {\mathrm{S}}_{{\mathrm{SH}}}$, i.e., $\Delta {\mathrm{S}}_{{\mathrm{SH}}} = {\mathrm{M}}_{{\mathrm{sz}}}\Delta {\mathrm{Z}}_{{\mathrm{DM}}}$. Here, we adopted the Zernike-polynomial-based wavefront reconstruction algorithm because it is less sensitive to noise and missing spots in the in vivo SHWS measurement^75^. This is particularly important when the intensity of the fluorescent guide star is low. Additionally, the ocular aberrations mainly consist of low-order aberrations^7^ (Fig. S13), making Zernike-based AO correction robust. It should be pointed out that this approach could induce additional calibration/correction errors by using an external interferometer, compared to the commonly used approach in AO ophthalmoscopy that measures the direct point-to-point mapping of the DM and SHWS. Quantitative comparison of the correction performance of these two methods will be studied in future work.

### Correction of system aberrations

Before measuring and correcting the aberrations of the mouse eye, we first compensated for the aberrations of our microscope system using a Zernike-mode-based sensorless AO algorithm^76^. We used the rhodamine 6G solution as the imaging sample, and the system correction refers to the DM pattern that maximizes the two-photon excited fluorescence intensity. To determine the optimal value for each Zernike mode, seven to nine different values of aberrations were applied to the DM, and the resulting fluorescence intensity was Gaussian fitted to find the centre of the curve. Typically, we measured up to 21 orders of Zernike modes (tip, tilt and defocus excluded) to correct aberrations of the microscope system.

### Correction of ocular aberrations

First, a reference spot pattern on SHWS S_ref_ was recorded by using the two-photon excited fluorescence signal of the rhodamine 6 G solution as the guide star with system aberrations corrected. To correct sample-induced aberration, we first measured the wavefront S_a_ of the descanned fluorescence signal in the mouse retina (GFP, GCaMP or Evans blue signal). Then, the sample-induced aberration was calculated as the relative displacements of SHWS spots $\Delta {\mathrm{S}}$, where $\Delta {\mathrm{S}} = {\mathrm{S}}_{\mathrm{a}} - {\mathrm{S}}_{{\mathrm{ref}}}$. Then, the corrective pattern added to the DM could be computed by solving a weighted minimization problem: $\Delta {\mathrm{Z}} = {{{\mathrm{arg}}\,{\mathrm{min}}}} \left\| {{\mathrm{W}}^{1/2}\left( {{\mathrm{M}}_{{\mathrm{sz}}}\Delta {\mathrm{Z}} + \Delta {\mathrm{S}}} \right)} \right\|^2 = - \left( {{\mathrm{W}}^{1/2}{\mathrm{M}}_{{\mathrm{sz}}}} \right)^+ {\mathrm{W}}^{1/2}\Delta {\mathrm{S,}}$ where $\left( \cdots \right)^ +$ represents the generalized matrix inverse and the weight parameter W is determined by the signal-to-background ratio of each spot on the SHWS. Because the two-photon excitation is well confined in the focal region, we achieve accurate measurement of ocular aberrations by using the nonlinear fluorescent guide star. As shown in Fig. S4, the AO correction converges in less than two iterations. The first iteration compensates for nearly all the aberrations and greatly improves the imaging resolution and signal intensity. The second iteration provides a sharper spot pattern and can further eliminate the residual wavefront errors. Therefore, in this work, we used at most two iterations of AO correction for each imaging position. As the wavefront was measured by integrating the fluorescent signals over a small field of view, the AO correction is insensitive to the respiration and heartbeat motion of mice. We found that under anaesthesia, the ocular aberration of mice was stable in more than 20 minutes (Fig. S13), and the AO correction remained effective during the imaging period.

### Animal preparation

Wild-type mice (ICR) and transgenic mice (Cx3Cr1-GFP) were used in this work. For structural imaging of RGCs and nerve fibres, ICR mice were injected intravitreally with AAV2-hsyn-GFP. For functional calcium imaging of RGCs, ICR mice were injected intravitreally with AAV9-hsyn-GCaMP6s. The in vivo retinal imaging experiments were conducted three weeks after virus injection^77,78^. Cx3Cr1-GFP transgenic mice were used for the imaging of microglia. To visualize retinal microvasculature, 200 µl of Evans blue (20 mg/ml) was injected intraperitoneally one hour before the imaging experiments. All the experimental procedures were approved by the Animal Ethics Committee of Hong Kong University of Science and Technology.

Before the imaging experiment, the mice were anaesthetized by intraperitoneal injection of a ketamine/xylazine cocktail. A metal bar was surgically fixed to the mouse skull and secured to a head-holding device with angle adjusters (MAG-2, NARISHIGE, Japan). The mouse pupil was dilated with one drop of 2.5% phenylephrine, and eye gel was applied (Genteal). Then, a 0-dioptre rigid contact lens (diameter 2.5 mm, OcuScience) matching the curvature of the mouse eyeball^79^ was placed on the mouse eye to maintain corneal hydration. The mouse was then placed on a translational stage of the AO-TPEFM system for in vivo retinal imaging. During the imaging experiment, mice were anaesthetized via inhalation of isoflurane gas (1–2% in oxygen).

### In vivo retinal imaging

For two-photon imaging and wavefront sensing, the 920 nm femtosecond laser was used to excite GFP, GCaMP or Evans blue signals with power less than 30 mW before the mouse eye. To measure the sample-induced aberration, the femtosecond laser was scanned over a FOV of 50–100 µm, and the excited fluorescence signal was integrated at the SHWS. The aberration measurement and correction took 0.1 to 2 s, depending on the fluorescence intensity. The correction pattern was applied to an isoplanatic volume of ~100 × 100 × 100 µm^3^. For structural imaging, the collection time was 1–2 s for each frame.

### Blood flow measurement

Evans blue was injected intraperitoneally to visualize the vasculature. A commonly used approach for blood flow measurement is by line scanning along the central line of the blood vessel^80^. To measure the velocity of an arbitrarily oriented blood vessel, the galvo X and Y scanners were scanned simultaneously along the direction of blood flow over a length of 10–20 µm at 0.5 kHz per line (Fig. S8). Then, the blood flow velocity was extracted using the Radon transform method^81^.

### Functional calcium imaging

For functional imaging experiments, a blue LED was used to stimulate the RGCs (Fig. 2a). The light from the LED was purified by a bandpass filter (FF02-447/60, Semrock) and directed to the mouse eye at a power level of ~60 μW/mm^2^. The duration and timing of the blue light flashes were controlled by AO-TPEFM software. In the recording of calcium transients, a short pulse (~10 ms) of blue light was flashed at the mouse eye at 10 s intervals^33^, while the two-photon fluorescence images were acquired at a frame rate of 3.8 Hz.

### Laser microsurgery

Laser microsurgery relies on a highly localized multiphoton ionization process^52^. A 740 nm femtosecond laser was directed to the microscope system to achieve imaging-guided laser microsurgery. To precisely ablate nerve fibres, first the aberration of the mouse eye was measured and corrected based on the GFP fluorescence guide star. Then, the 740 nm laser with an average power of 350 mW was focused at the nerve fibres for 1–4 s, and the new fluorescence signal produced by the laser ablation process was used as the feedback to control the exposure time of the 740 nm laser and evaluate the microsurgery dimensions^51^. To study axon degeneration following laser axotomy, the ablated region was imaged repeatedly for a few days.

### Activation and imaging of microglia

2 µl of N-methyl-D-aspartic acid (NMDA, Sigma, 10 mM in PBS) was injected intravitreally into Cx3Cr1-GFP mice to induce retinal inflammation^43^, and in vivo imaging was conducted before NMDA injection and four and eight hours after NMDA injection.

### Image analysis

All images were processed with ImageJ (NIH). The image scale on the mouse retina was simulated using the schematic eye^82^, and each degree corresponds to approximately 31 μm. The spatial resolution of AO-TPEFM for retinal imaging was estimated from the cross-sections of the dendritic processes of microglia (Figs. 1e, f and S7), and the mean value and standard deviation from six locations were reported. For the mosaic images of RGCs, blood vessels or microglia, the sub-images were assembled using the ‘MosaicJ’ plugin in ImageJ. To remove the inter-frame motion artefacts, imaging registration was performed using the ‘TurboReg’ or ‘StackReg’ plugin in ImageJ^83^. Intra-frame distortion was not observed, probably due to the short acquisition time of fluorescence images (~1 s). For the calcium imaging data, the average fluorescence intensity in the region of interest was measured, and the calcium response was calculated by $\frac{{\Delta F}}{F}\left( t \right) = \frac{{F\left( t \right) - F_0}}{{F_0}}$, where *F*_0_ is the baseline signal. The mean response was calculated by averaging several individual responses and was fitted with an exponential function to calculate the decay half-lives.

## Supplementary information

Supplementary Information

Video S1

Video S2

Video S3

Video S4

Video S5
