# Supplementary material for: Adaptive optics two-photon microscopy enables near-diffraction-limited and functional retinal imaging in vivo
Source: Light Sci Appl. 2020 May 6;9:79. doi: 10.1038/s41377-020-0317-9 (PMC7203252; doi:10.1038/s41377-020-0317-9)
Supplement: Supplementary file 1 — Supplementary Information [file 41377_2020_317_MOESM1_ESM.docx]

**Supplementary information for**

Adaptive optics two-photon microscopy enables near-diffraction-limited and functional retinal imaging *in vivo*

Zhongya Qin^1,6^, Sicong He^1,6^, Chao Yang^2^, Jasmine Sum-Yee Yung^5^, Congping Chen^1^, Christopher K.S. Leung^5^, Kai Liu^2,3,4^ and Jianan Y. Qu^1,3,4,*^

^1^Department of Electronic and Computer Engineering, The Hong Kong University of Science and Technology, Clear Water Bay, Kowloon, Hong Kong, China

^2^Division of Life Science, The Hong Kong University of Science and Technology, Clear Water Bay, Kowloon, Hong Kong, China

^3^State Key Laboratory of Molecular Neuroscience, The Hong Kong University of Science and Technology, Clear Water Bay, Kowloon, Hong Kong, China

^4^Center of Systems Biology and Human Health, The Hong Kong University of Science and Technology, Clear Water Bay, Kowloon, Hong Kong, China

^5^Department of Ophthalmology and Visual Sciences, The Chinese University of Hong Kong, Hong Kong, China

^6^These authors contributed equally to this work

^*^Corresponding author: [eequ@ust.hk](mailto:eequ@ust.hk) (J.Y.Q.)

**
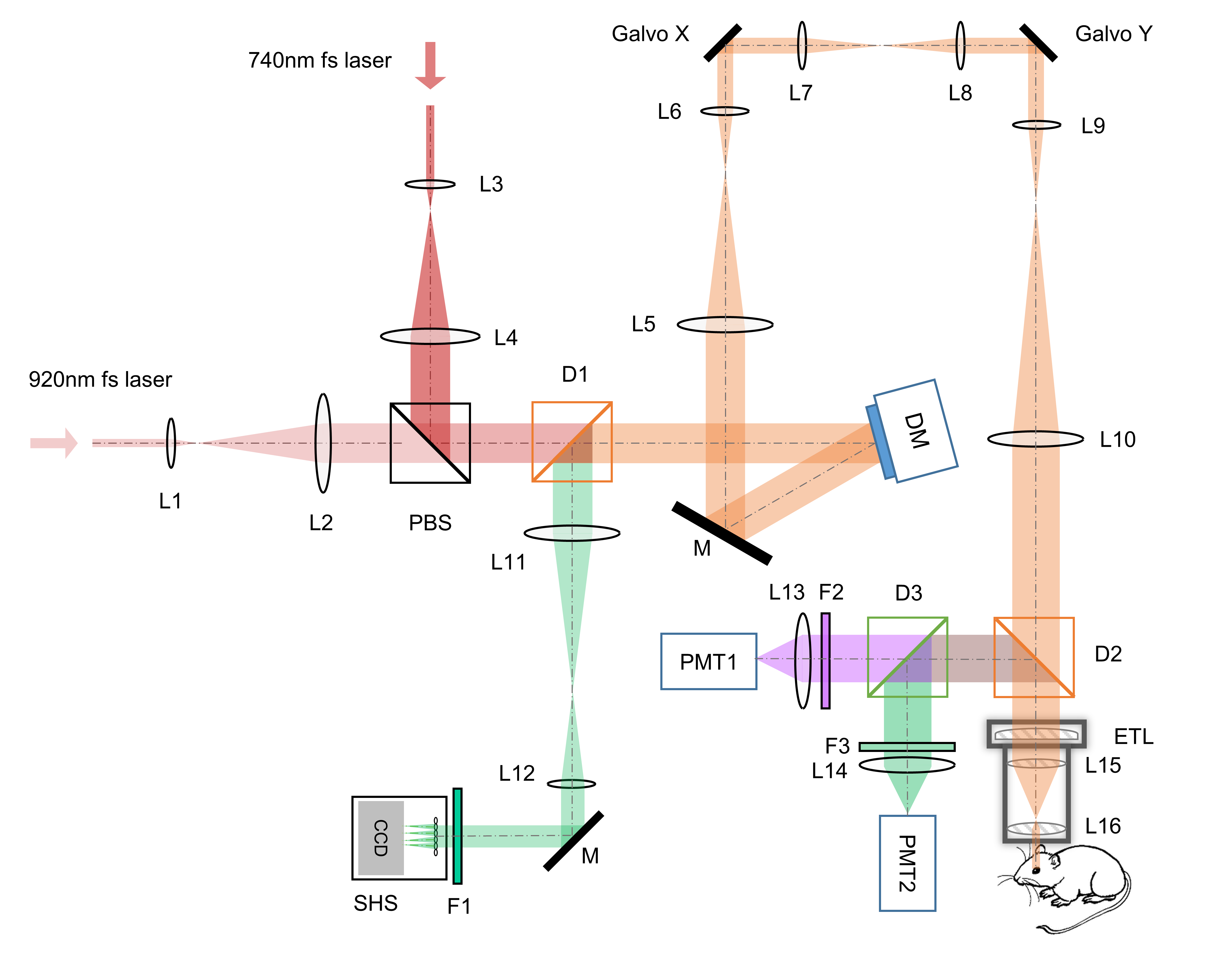
**

**Fig. S1 Optical layout of the AO-TPEFM system for mouse retinal imaging.** To minimize pupil shift, five 4-f telescopes were applied to conjugate the deformable mirror (DM), Shack-Hartmann wavefront sensor (SHWS), galvanometer scanning mirrors, electrically tunable lens (ETL) and the mouse cornea. L, lens; PBS, polarizing beamsplitter, D, dichroic mirror; M, mirror.

**
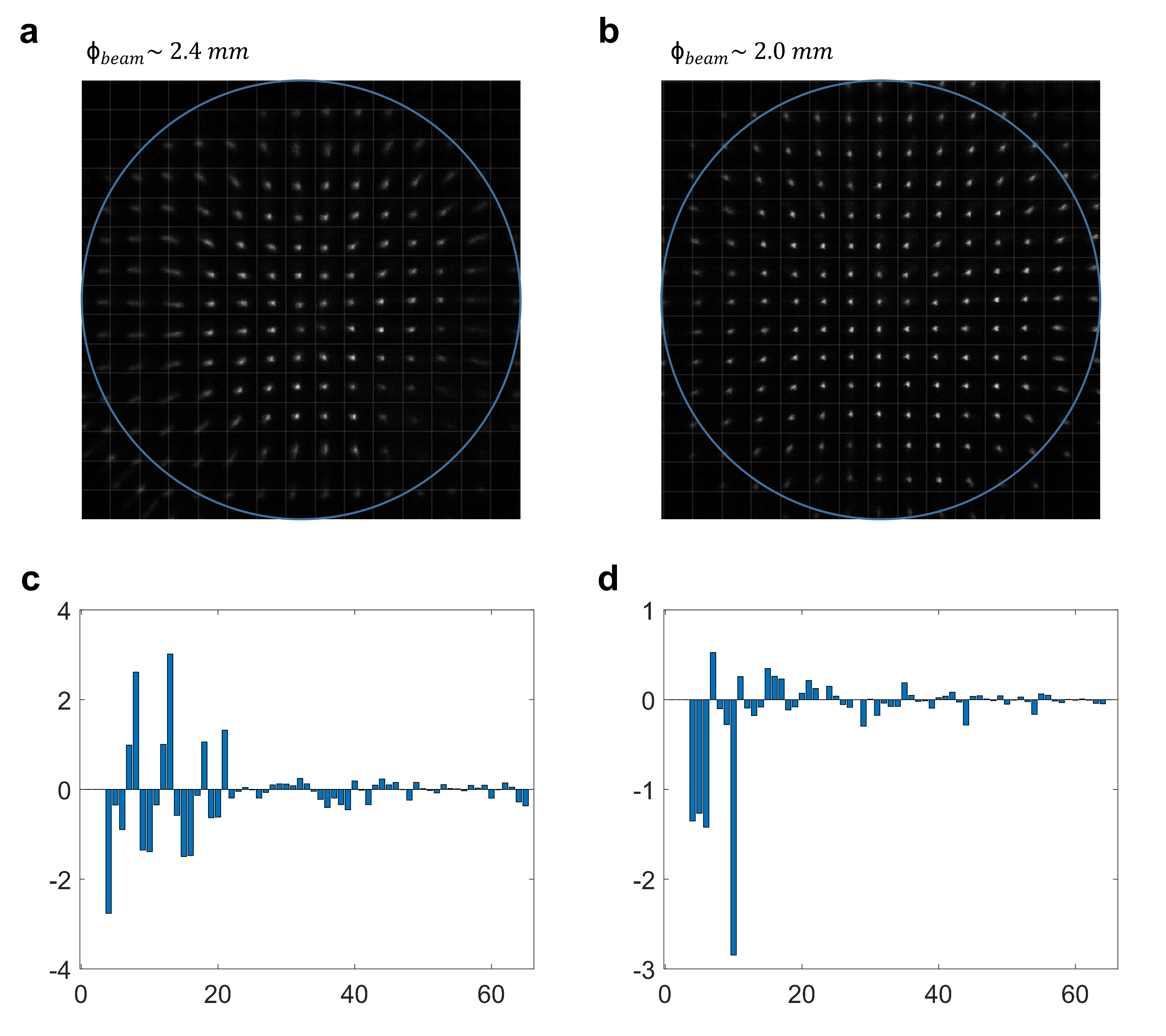
**

**Fig. S2 An optimal pupil size is required for accurate wavefront sensing.** (a) The spot pattern on the SHWS for a $\phi$ 2.4 mm pupil size. (b) The amplitudes of the Zernike modes in a modal decomposition of the wavefronts shown in (a). (c) The spot pattern on the SHWS for a $\phi$ 2.0 mm pupil size. (d) The amplitudes of the Zernike modes in a modal decomposition of the wavefronts shown in (c).


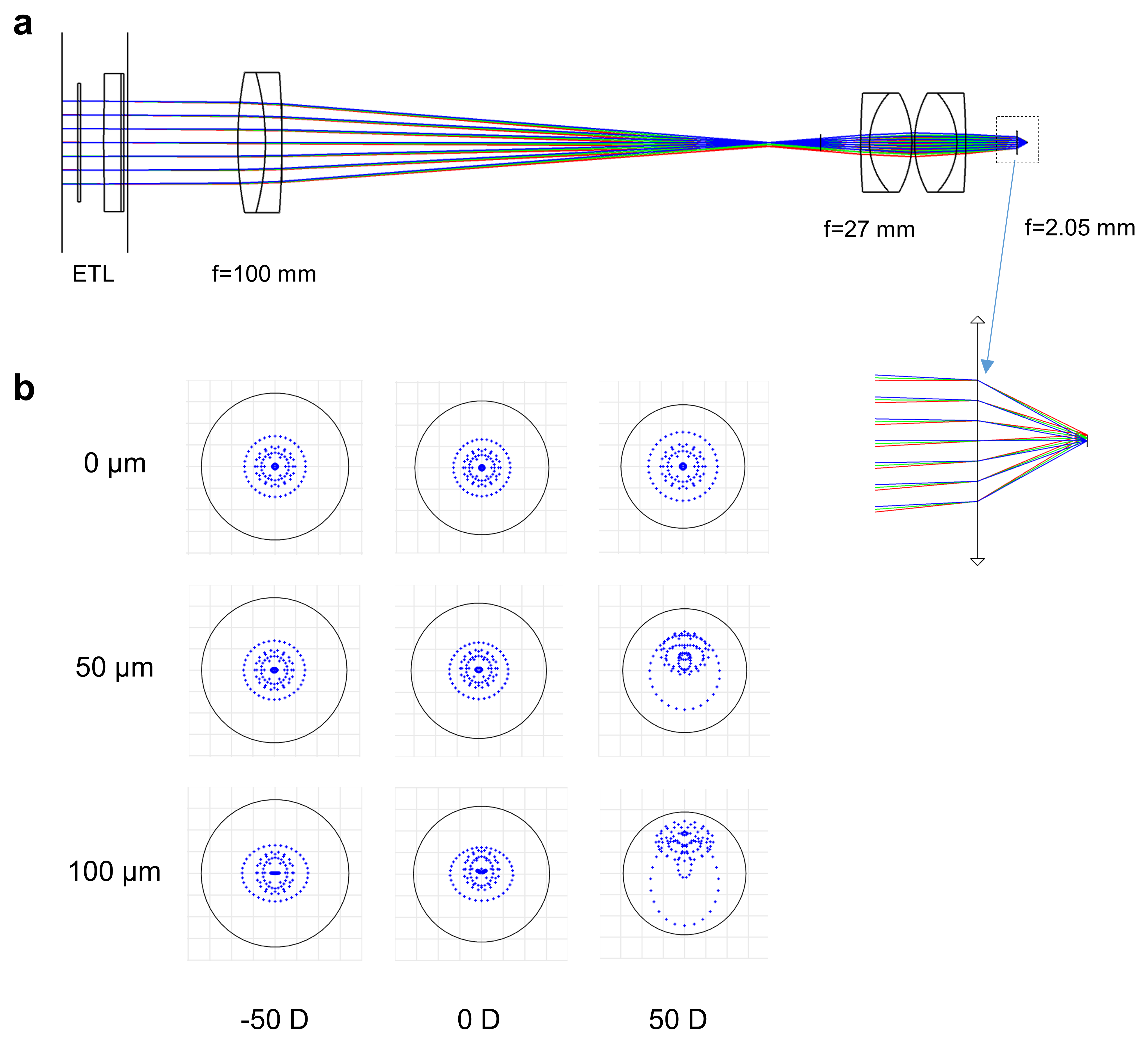


**Fig. S3 Simulation of the add-on module for retinal imaging.** (a) Optical layout of the add-on module. For easy incorporation into a standard two-photon microscope, the whole length of the module is designed to be < 180 mm and the entrance pupil diameter is about 15 mm. The enlarged image shows that the excitation laser beam is stable at the pupil of the mouse eye across the scanning range of 200 μm. (b) Spot diagrams for the nine configurations analyzed in the retinal plane, over a 200 μm FOV for the focal range from -50 to 50 diopters. All the configurations are diffraction limited for the 920 nm wavelength and the radii of the airy disk (black circle) are 1.36, 1.25 and 1.15 μm for -50, 0 and 50 diopters respectively (wavelength: 920 nm).

**
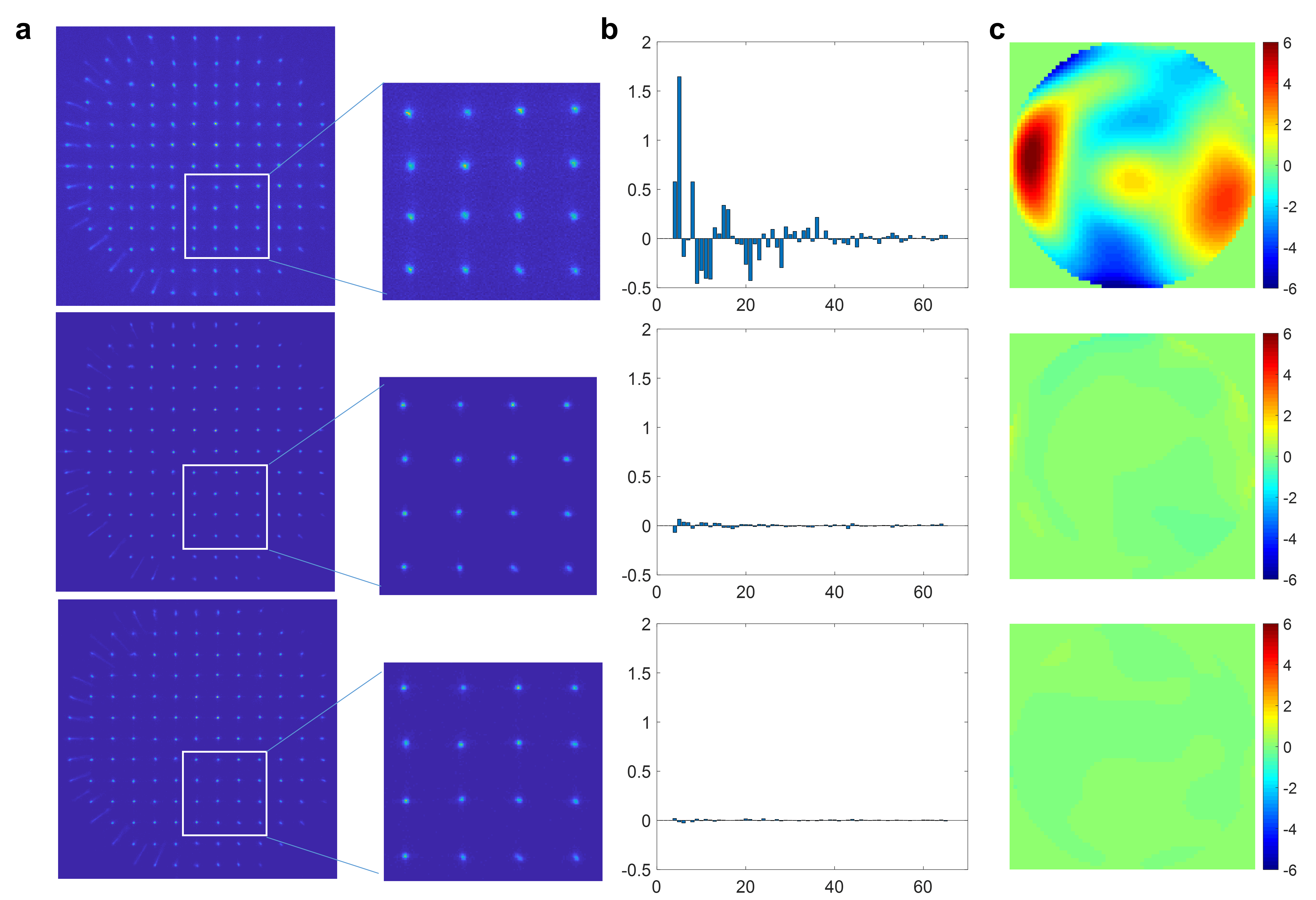
**

**Fig. S4 AO correction in the closed-loop configuration.** (a) Spot pattern on the SHWS in three iterations of AO correction. Right: enlarged images of the white box region show that spots on the SHWS become much sharper after the first iteration. (b) Changes of the DM in the form of Zernike coefficients in the three iterations. As can be seen, the AO correction converges after two iterations. Therefore, in this work, we applied two iterations of AO correction for each imaging location. (c) Wavefront changes of the DM corresponding to (b). Unit: μm.

**
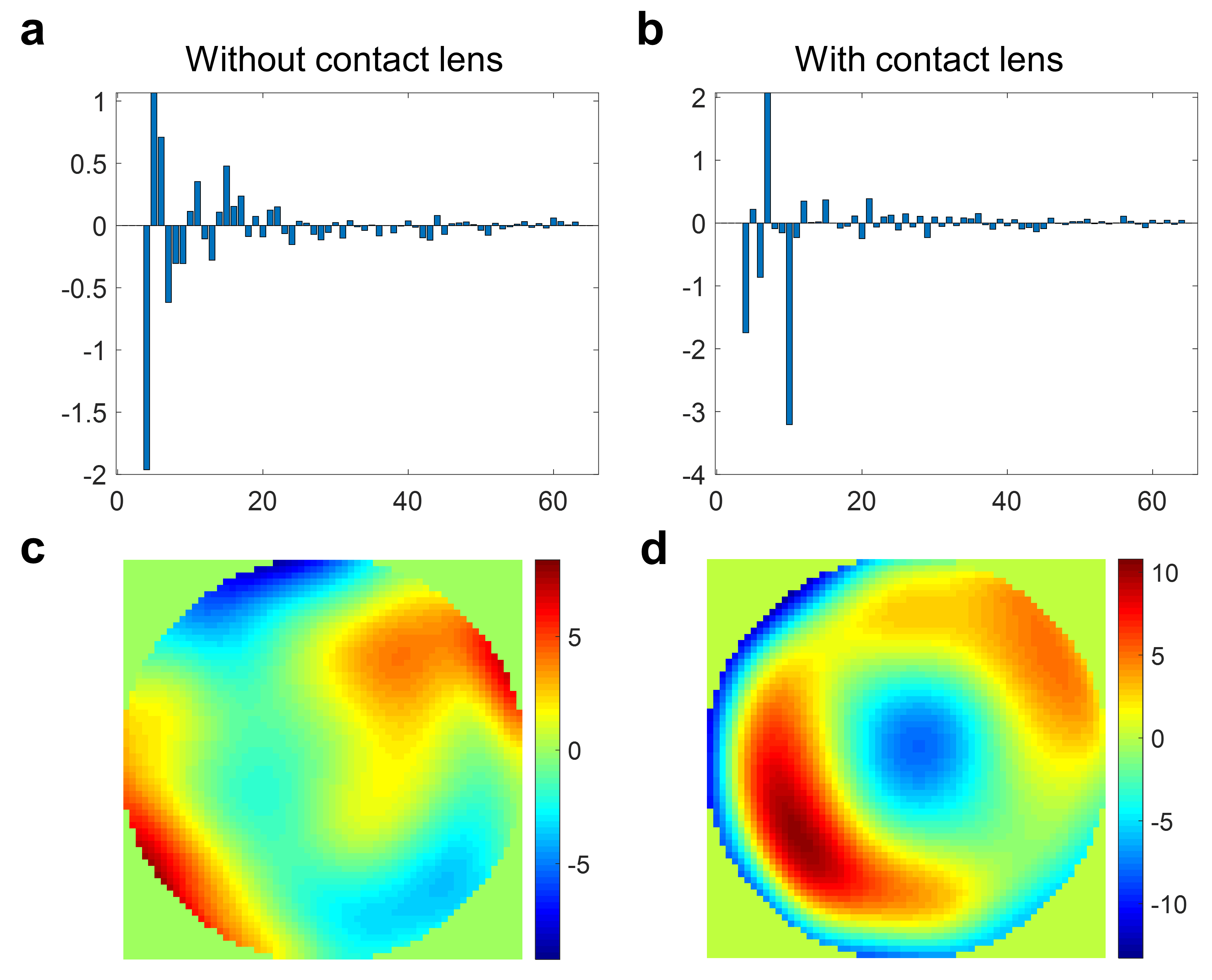
**

**Fig. S5 Effect of the contact lens on the aberration measurement.** (a-b) Aberrations of the mouse eye without (a) and with (b) a contact lens in the form of Zernike polynomials. (c-d) The wavefront maps corresponding to (a-b). Unit: μm.

**
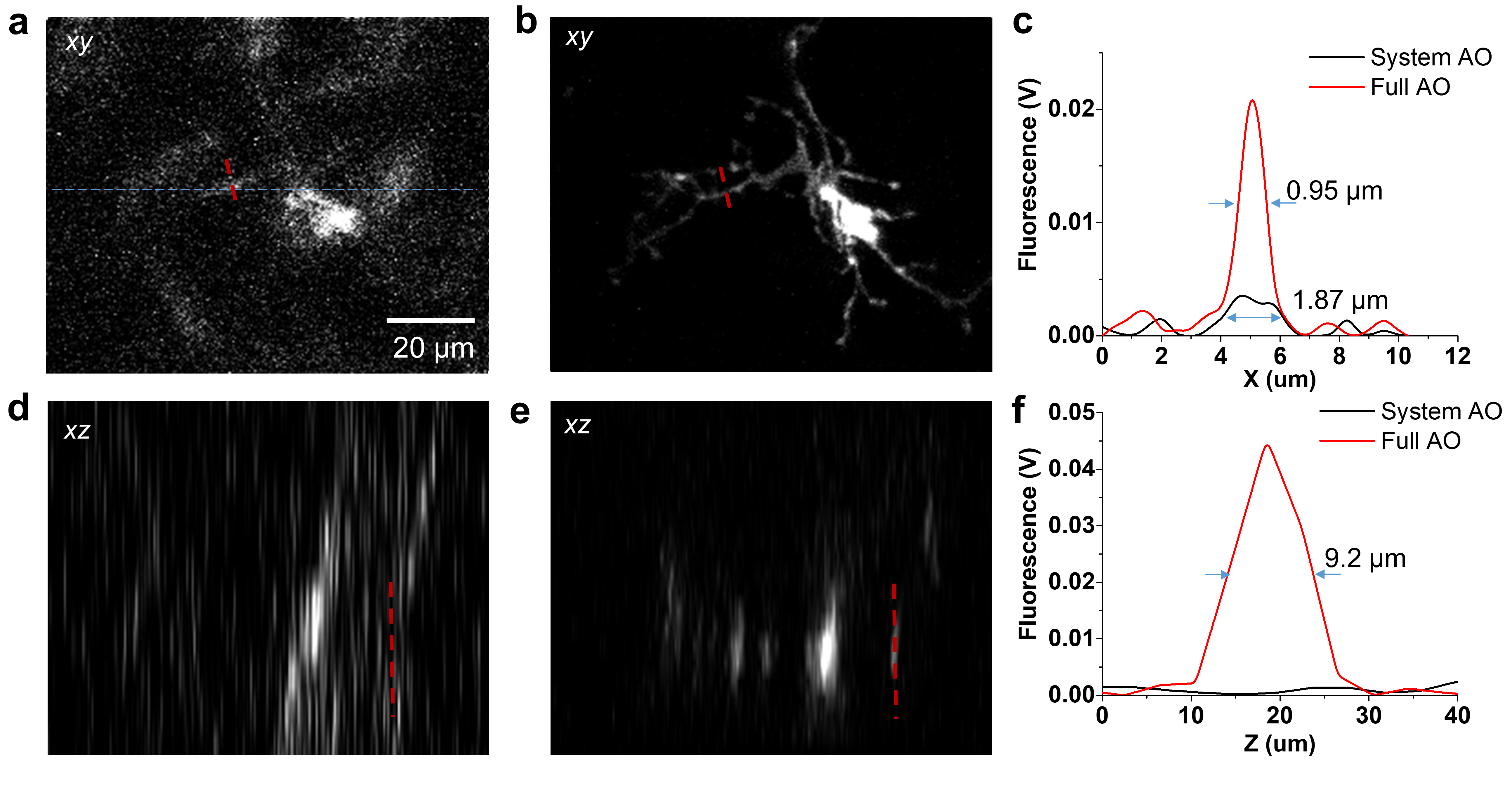
**

**Fig. S6 Estimation of imaging resolution using the fine processes of microglia.** (a-b) The x-y maximum-intensity projection (MIP) images of microglia with system (a) and full (b) AO correction. (c) The transverse cross sections of the fine process of microglia (red dashed line in (a-b)) was used to estimate the lateral imaging resolution before and after AO correction. (d-e) The x-z orthoslice through the plane defined by the blue dashed line in (a) with system (d) and full (e) AO correction. (f) The axial cross section of point objects on the microglial process (red dashed line in (d-e)) was used to estimate the axial imaging resolution before and after AO correction. Because the signal-to-background ratio is too low with only system AO correction, the axial resolution cannot be accurately determined.

**
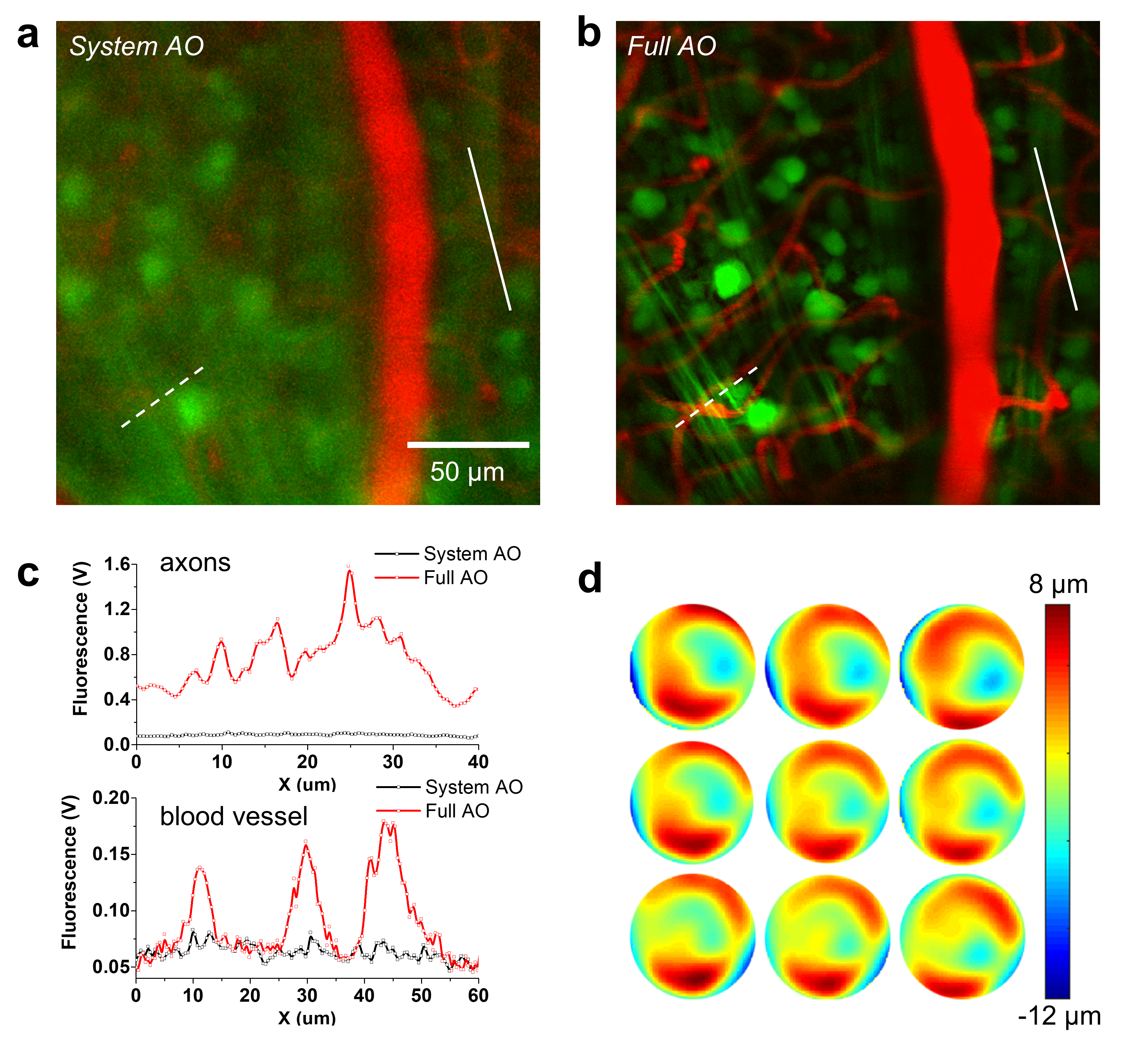
**

**Fig. S7 AO-TPEFM enables two-color imaging of RGCs and blood vessels.** (a-b) Mosaic projection TPEF images of RGCs (green) and blood vessels (red) with system (a) and full (b) AO correction. (c) Signal profiles for a comparison of fluorescence intensity with system (red line) and full (black line) AO correction. Top: fluorescence intensity profile of RGCs along the white dashed line in (a-b). Bottom: fluorescence intensity profile of blood vessels along the white solid line in (a-b). (d) The full AO correction wavefront map used in (b).

**
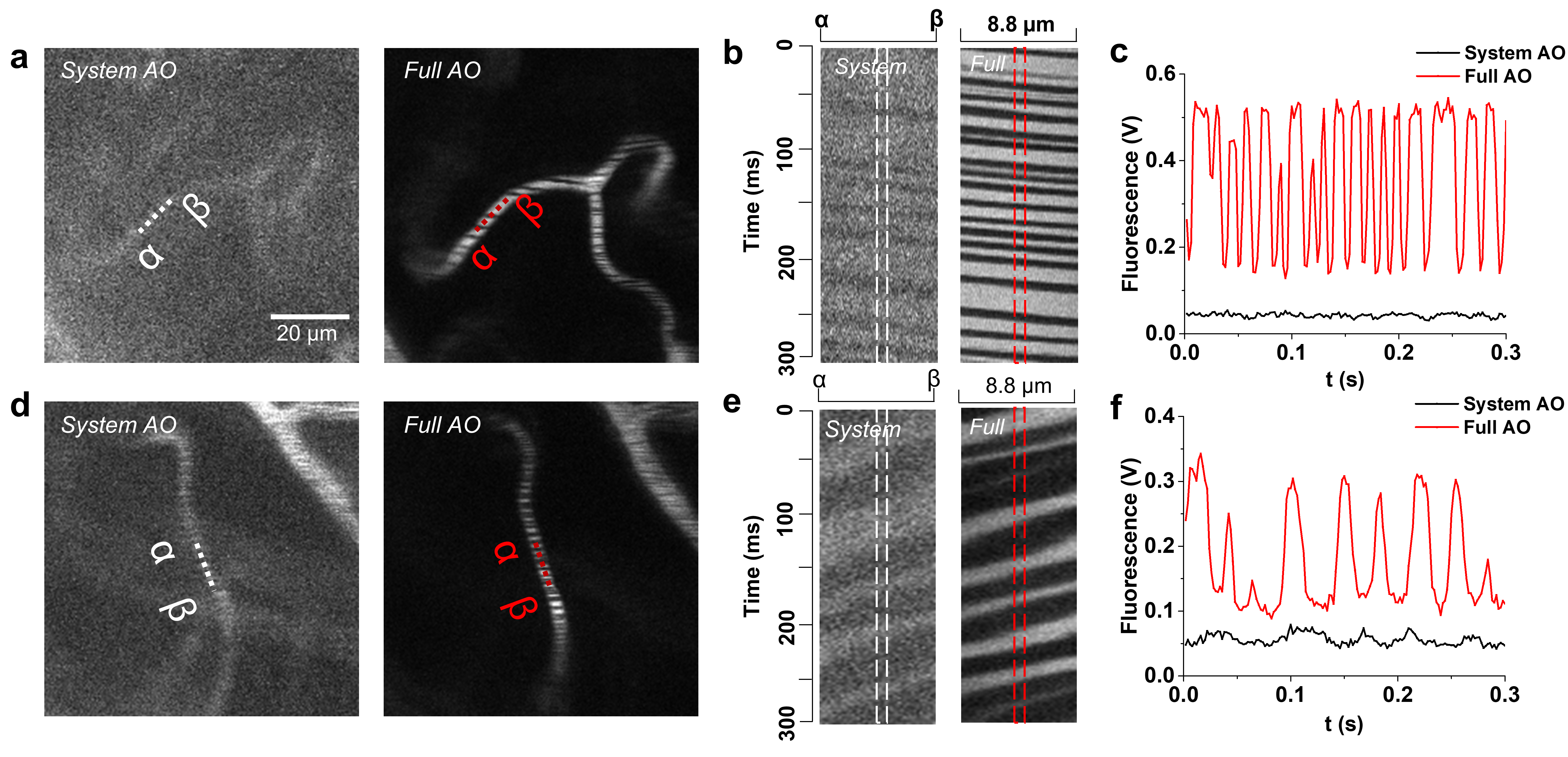
**

**Fig. S8 AO-TPEFM enables high-resolution imaging of blood vessels and precise measurement of blood flow velocity.** (a) Imaging of microvasculature with system and full AO correction. (b) Space-time plot of the line scan signals along the capillary marked by the dashed line α-β in (a) showing the flux of red blood cells. (c) Fluorescence intensity comparison of system and full AO correction along the dashed lines in (b). The average blood flow velocity was estimated to be 0.87 mm/s using radon transform. (d-f) Another example showing that AO greatly increased the fluorescence intensity and imaging resolution. The average blood flow velocity was 0.30 mm/s.

**
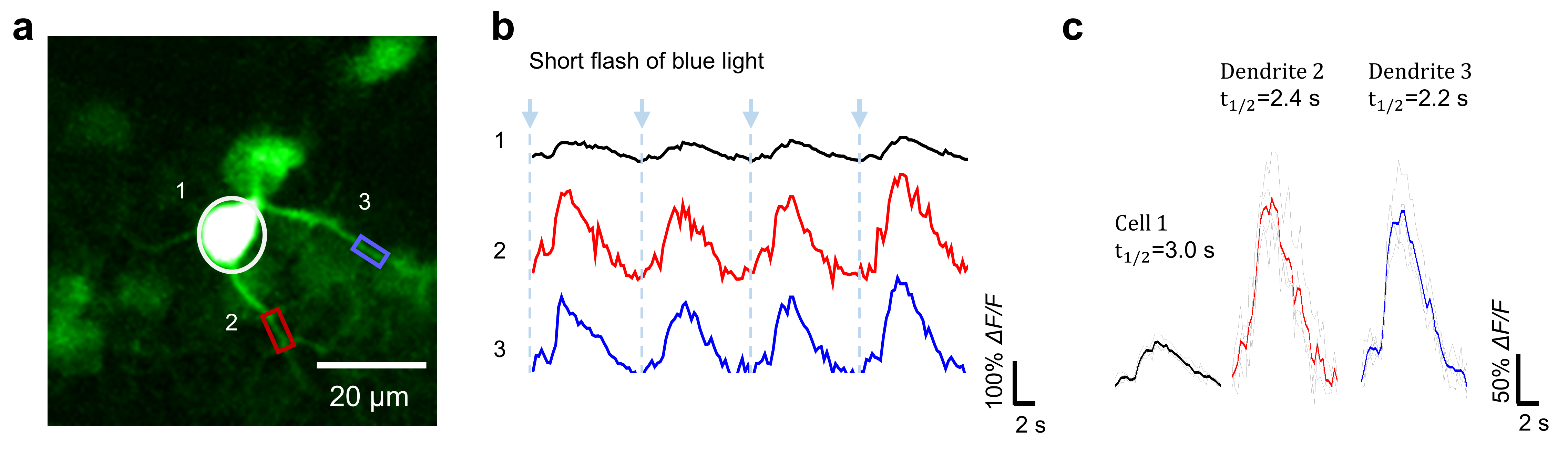
**

**Fig. S9 Simultaneous calcium imaging of RGC somas and dendrites.** (a) Images of the GCaMP-labeled RGCs with full AO correction. (b) Calcium traces of the RGC soma and dendrites marked in (a). (c) The changes in fluorescence intensity (∆f/f) and decay half-lives of the soma and dendrites.

**
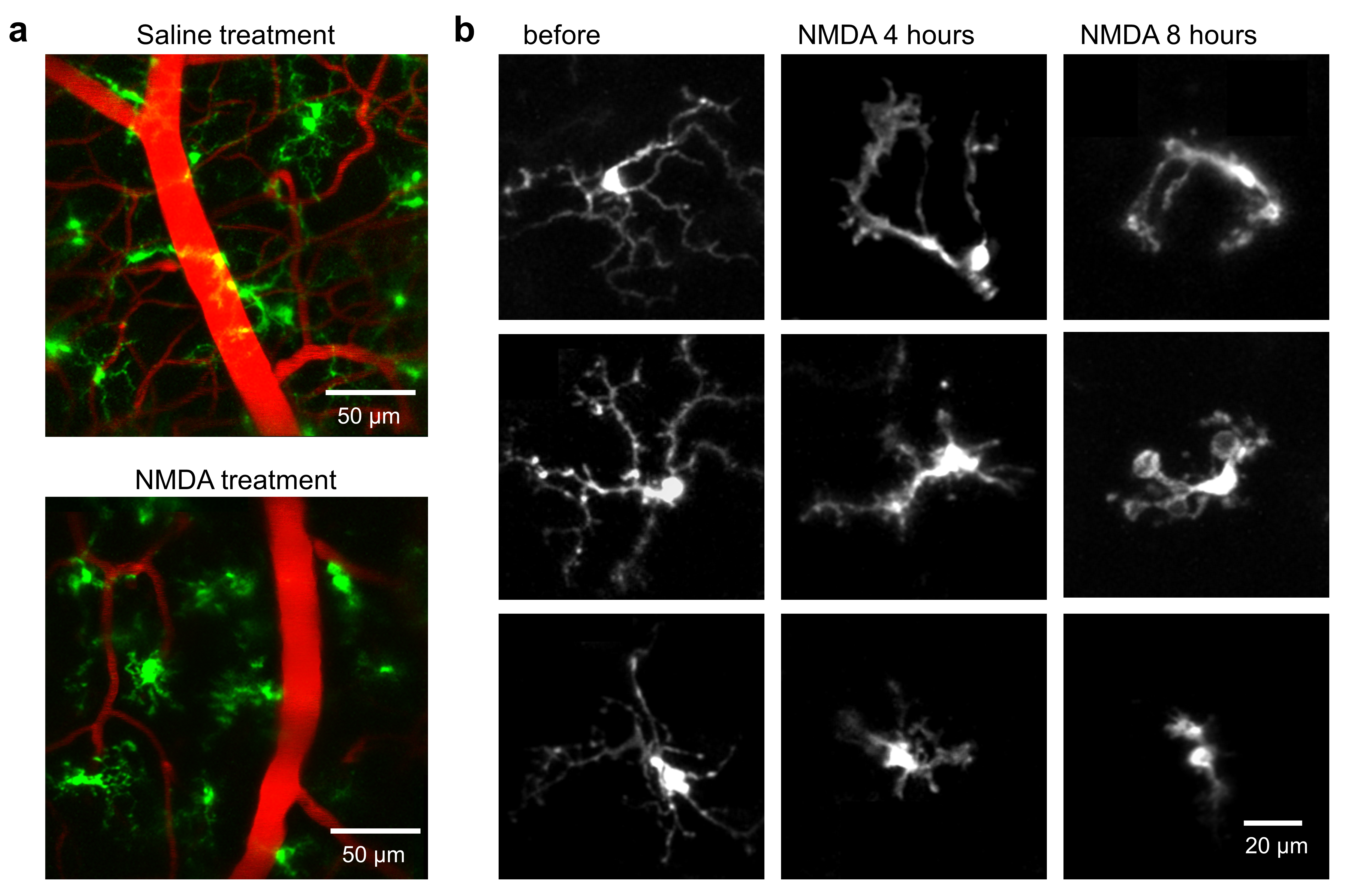
**

**Fig. S10 Structural analysis of microglia in the NMDA-administrated mouse retina. (a)** In the control eye injected with saline, the “resting” microglia show elaborate ramifications, while the “activated” microglia in the NMDA-injected eye exhibit more amoeboid morphologies with retracted processes. Images were captured eight hours after saline/NMDA injection. (b) Representative images of microglia morphology before NMDA treatment, and four and eight hours after NMDA treatment.

**
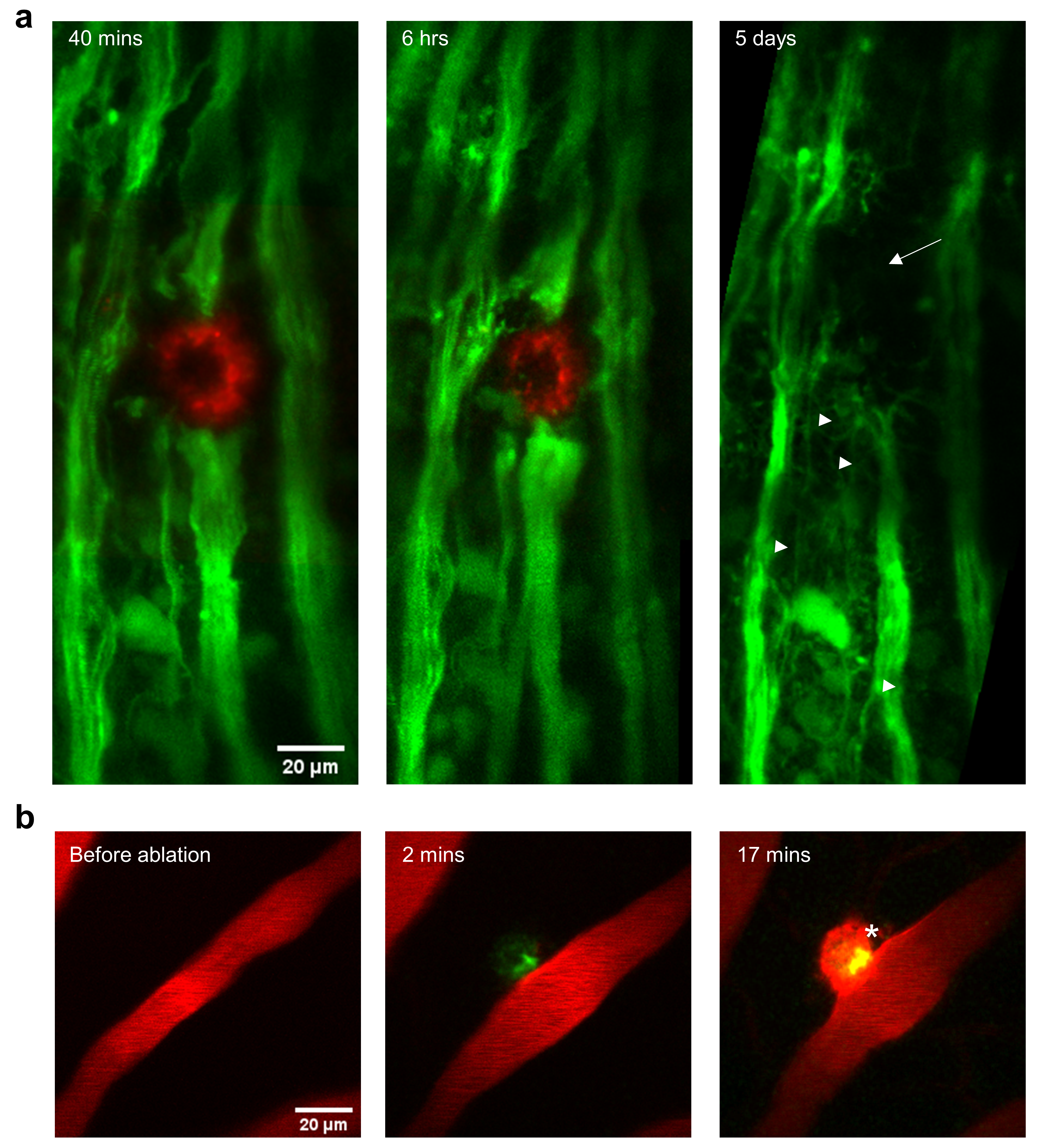
**

**Fig. S11 AO-TPEFM enables controllable injury to retinal axons and vascular structures.** (a) Demonstration of a large lesion on a number of nerve fiber bundles. The white arrow shows the retraction of distal axons and white arrowheads mark the degeneration of proximal axons. Green: GFP-labeled retinal neurons; red: fluorescence signal produced by laser ablation. (b) Demonstration of laser injury to retinal vasculature. Red: retinal blood vessels labeled with Evans blue; green: fluorescence signal produced by laser ablation. The leakage of fluorescent dye was marked by the star, which indicates the injury to blood vessel.


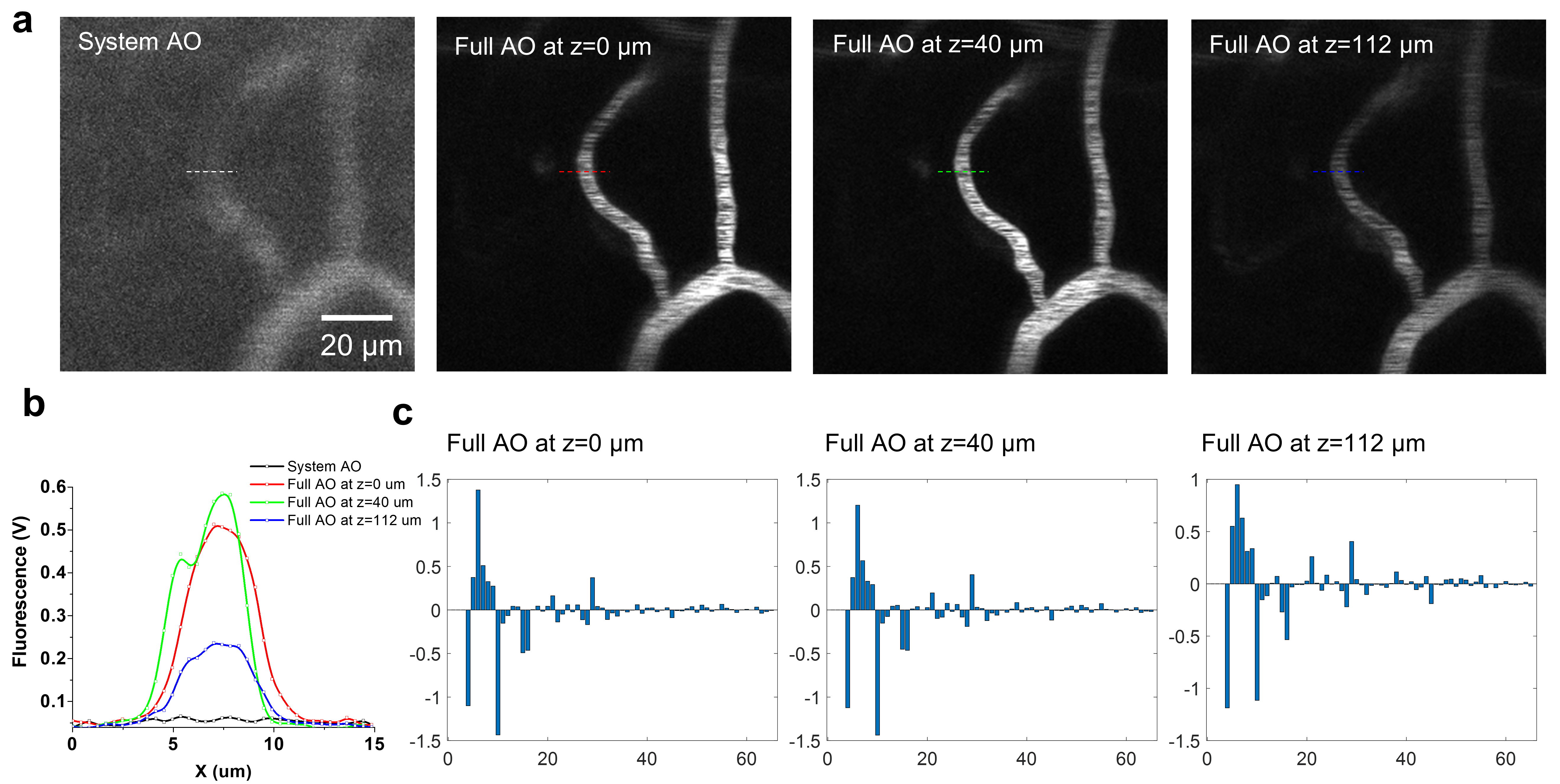


**Fig. S12 Ocular aberration varies with the imaging depth.** (a) Two-photon images of blood vessels at the ganglion cell layer (GCL) using system AO correction and full AO correction at different depths. (b) Comparison of fluorescence intensity along the dashed lines in (a). This clearly shows that using the wavefront measured at the photoreceptor layer (> 100 μm away from GCL) is not the best correction for the GCL. (c) Aberration measured in distinct retinal layers in the form of Zernike polynomials.


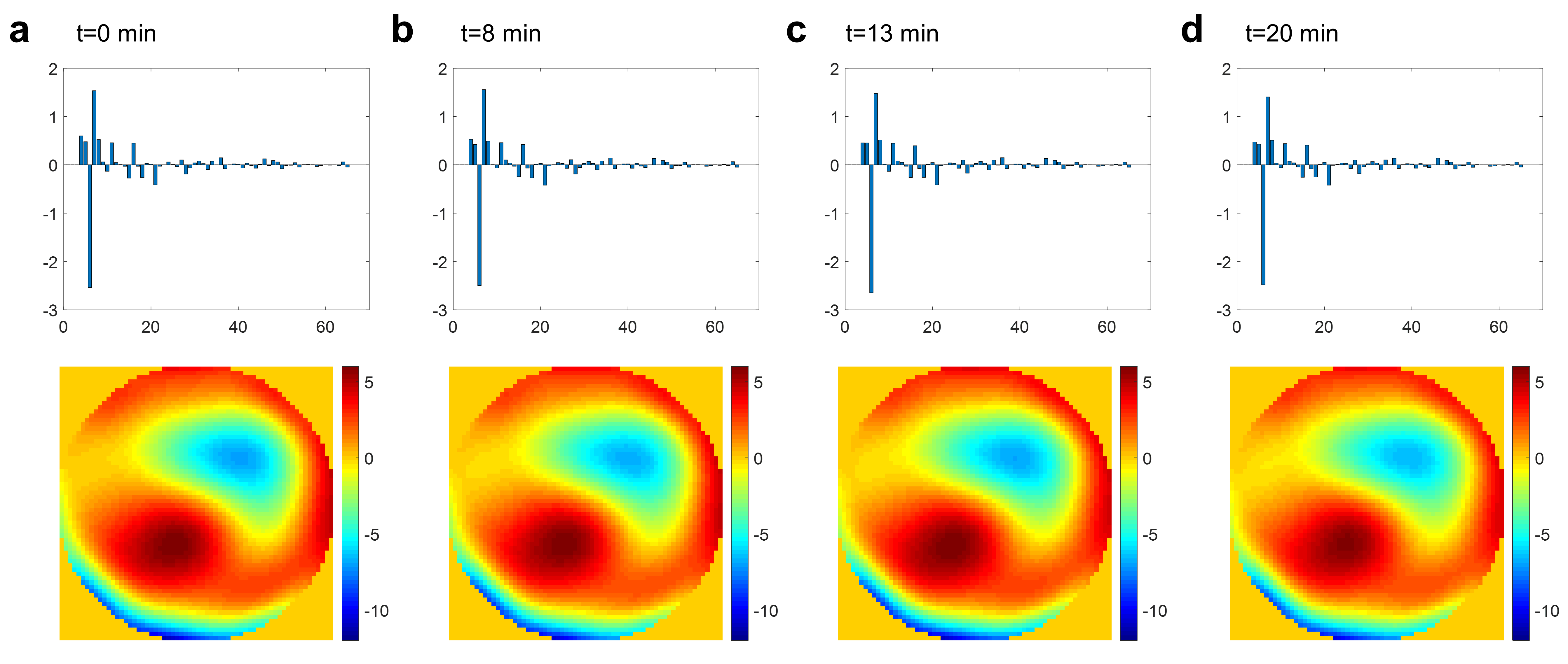


**Fig. S13 The ocular aberration was stable within 20 minutes.** (a-d) Aberration of mouse eye measured in the same position at (a) t=0 min, (b) t=8 min, (c) t=13 min and (d) t=20 min. GFP signals were used as guide stars. Upper row: aberration in the form of Zernike polynomials; bottom row: the corresponding wavefront map. Units: μm.
